# Gender Disparity between Cutaneous and Non-Cutaneous Manifestations of Lyme Borreliosis

**DOI:** 10.1371/journal.pone.0064110

**Published:** 2013-05-30

**Authors:** Franc Strle, Gary P. Wormser, Paul Mead, Kanthi Dhaduvai, Michael V. Longo, Omosalewa Adenikinju, Sandeep Soman, Yodit Tefera, Vera Maraspin, Stanka Lotrič-Furlan, Katarina Ogrinc, Jože Cimperman, Eva Ružić-Sabljić, Daša Stupica

**Affiliations:** 1 Department of Infectious Diseases, University Medical Center Ljubljana, Ljubljana, Slovenia; 2 Division of Infectious Diseases, New York Medical College, Valhalla, New York, United States of America; 3 National Center for Emerging and Zoonotic Infectious Diseases, Centers for Disease Control and Prevention, Fort Collins, Colorado, United States of America; 4 Institute for Microbiology and Immunology, Medical Faculty Ljubljana, Ljubljana, Slovenia; University of Toledo School of Medicine, United States of America

## Abstract

Cutaneous manifestations of Lyme borreliosis in Europe include erythema migrans (EM) and acrodermatitis chronica atrophicans (ACA); the most common non-cutaneous manifestations are Lyme neuroborreliosis (LNB) and Lyme arthritis. The purpose of this study was to evaluate the gender distribution of patients with these clinical manifestations of Lyme borreliosis. Data on gender were obtained from the clinical records of patients with Lyme borreliosis aged ≥15 years who had been evaluated at the University Medical Center Ljubljana, Ljubljana, Slovenia.

Among 10,539 patients diagnosed with EM, 6,245 (59.3%) were female and among 506 ACA patients 347 (68.6%) were female. In contrast, among the 60 patients with Lyme arthritis only 15 (25%) were female (p<0.0001 for the comparison of gender with EM or ACA) and among the 130 patients with LNB only 51 (39.2%) were females (p<0.0001for the comparison of gender with EM or ACA). Although the proportion that was female in the LNB group was greater than that of patients with Lyme arthritis, this difference did not reach statistical significance (p = 0.10). Although older individuals are more likely to be female in the general Slovenian population, the age of patients with cutaneous versus non-cutaneous manifestations was not the explanation for the observed differences in gender.

In conclusion, patients with cutaneous manifestations of Lyme borreliosis were predominantly female, whereas those with non-cutaneous manifestations were predominantly male. This provocative finding is unexplained but may have direct relevance to the pathogenesis of Lyme borreliosis.

## Introduction

Lyme borreliosis is transmitted by the bite of *Ixodes* ticks infected with *Borrelia burgdorferi* sensu lato (the term “sensu lato” refers to all of the species of Lyme borrelia) [1]. In the United States there is a male predominance of cases [2], [3]. This is not necessarily true in Europe where in some countries such as Germany there is a female predominance [4], [5]. It has been presumed that the explanation for the unequal gender distribution is that the likelihood of tick exposure is greater for males in the United States and greater for females in certain European countries.

In the United States, only *B. burgdorferi* sensu stricto (the term “sensu stricto” specifically refers to one particular species of Lyme borrelia) causes Lyme borreliosis whereas in Europe the majority of cases of Lyme borreliosis are caused by *B. afzelii* and *B. garinii*, rather than *B. burgdorferi* sensu stricto [1]. Consequently, there are a greater variety of clinical manifestations of Lyme borreliosis in Europe compared with the United States.

In this study we have evaluated the gender distribution according to clinical manifestation among patients with Lyme borreliosis evaluated at the University Medical Center Ljubljana in Ljubljana, Slovenia. Slovenia is a small central European country with 2 million inhabitants. From 2008 to 2011, the annual incidence of Lyme borreliosis was ≥250 cases/100,000 inhabitants [6].

## Methods

### Patient Population and Serologic Testing Methods

Clinical records were reviewed of patients with Lyme borreliosis aged ≥15 years who were evaluated at the University Medical Center Ljubljana, Ljubljana, Slovenia during 1990–2012. To account for changes in clinic procedures over time, data extraction was limited to periods when information on a specific manifestation was systematically collected, resulting in somewhat different time frames for the different manifestations. Erythema migrans (EM) was defined according to the Centers for Disease Control and Prevention criteria as an erythematous skin lesion of ≥5 cm in diameter [7]. EM patients diagnosed during the period 1990 to 2009 were included in the analysis.

Patients with acrodermatitis chronica atrophicans (ACA) were required to have a characteristic clinical picture, supportive histologic findings, and serum IgG antibody to borrelial antigens. ACA patients diagnosed from 1990 to July 2012 were included in the analysis. For patients with ACA, different serological methods were used over the 23 year time frame. Initially, IgG seropositivity in serum to *B. burgdorferi* sensu lato required either a positive immunofluorescence assay [8] or a positive enzyme linked immunosorbent assay with a positive supplemental IgG immunoblot [9]. In the past few years testing has been based on an indirect chemiluminescence immunoassay (LIAISON®, Diasorin, Italy) using the VlsE (variable major protein-like sequence expressed)recombinant antigen [10], following the manufacturer's recommendations.

Patients with Lyme arthritis had objective joint swelling in one or a few large joints, serum IgG antibody to borrelial antigens and no alternative explanation for the arthritis. The analysis included Lyme arthritis patients diagnosed between 2001 and July 2012. For patients with Lyme arthritis, IgG antibodies to *B. burgdorferi* sensu lato in serum were determined byan indirect chemiluminescence immunoassay (LIAISON®, Diasorin, Italy) using recombinant VlsE, following the manufacturer's recommendations.

Patients diagnosed with Lyme neuroborreliosis (LNB) were required to have cerebrospinal fluid (CSF) pleocytosis and at least one of the following: a) concomitant EM, b) isolation of *B. burgdorferi* sensu lato from CSF, or c) evidence of intrathecal synthesis of antibody to borrelial antigens. Patients diagnosed between October 2005 and July 2012 were included in the analysis. For patients with LNB, antibodies to *B. burgdorferi* sensu lato in serum and CSF were detected byan indirect chemiluminescence immunoassay (LIAISON®, Diasorin, Italy) using recombinant outer surface protein C (OspC) and recombinant VlsE as antigens for IgM antibody, and recombinant VlsE as the antigen for IgG antibody. Intrathecal synthesis of borrelial antibodies was determined using the approach described by Reiber and Peter: antibody index values >1.4 were interpreted as indicating production of intrathecal borrelial antibody [11]. In some patients intrathecal antibody synthesis to *B. burgdorferi* sensu lato was also determined using the IDEA™ Lyme Neuroborreliosis kit (DakoCytomation, Cambridgeshire, UK) that uses the flagellin antigen from *B. afzelii*; an index of ≥0.3 indicates intrathecal synthesis of specific borrelial antibodies with this assay.

### Analysis

As in many countries, the overall sex ratio in Slovenia varies from male biased among younger age groups to female biased among older age groups [12]. Some differences in the sex ratio are therefore inherent when comparing groups of patients with different average ages. To correct for this, sex ratios for each clinical group were normalized using the direct standardization method, 5-year age groupings, and data for the Slovenian population for the year 2000 [12]. Statistical comparisons between groups were made after applying the age-adjusted sex ratio to the number of patients in each group and comparing the distributions by the chi-square test [13], [14].

### Ethics Statement

The investigation was conducted according to the principles expressed in the Declaration of Helsinki. The study was approved by the National Medical Ethics Committee of the Republic of Slovenia (No 117/11/12). The Ethics Committee waived the need for written informed consent.

## Results

Among the 10,539 Slovenian patients ≥15 years old diagnosed with EM from 1990 to 2009, 6,245 (59.3%) were female (Table 1). A similar female predominance of 57.7% was found among the subgroup of 580 patients with multiple EM skin lesions. Among the 506 ACA patients ≥15 years old diagnosed from 1990 to July 2012, 347 (68.6%) were female. Although the proportion of females was greater among patients with ACA than EM, this difference did not reach statistical significance after correcting for differences in age (*X^2^* = 1.2, p = 0.27).

**Table 1:** Gender and Age of Slovenian Patients with Lyme Borreliosis According to Clinical Manifestation.

Manifestation	Years When Diagnosed	Number	Age in Years Median (range)	Number of Females (%)	Age of Females in Years Median (range)	Number of Males (%)	Age of Males in Years Median (range)	Age Adjusted Percent Female
Erythema migrans	1990–2009	10,539	48 (15–94)	6245 (59.3)	50 (15–94)	4294 (40.7)	45 (15–91)	56.9
Acrodermatitis chronica atrophicans	1990–2012	506	64 (15–91)	347 (68.6)	65 (15–91)	159 (31.4)	62 (21–89)	62.5
Lyme neuroborreliosis	2005–2012	130	52.5 (15–79)	51 (39.2)	57.5 (19–78)	79 (60.8)	48 (15–79)	34.4
Lyme arthritis	2001–2012	60	48.5 (16–79)	15 (25.0)	50 (26–70)	45 (75.0)	46 (16–79)	21.0

In contrast, there was a striking male predominance among patients with Lyme arthritis. Among the 60 patients with Lyme arthritis ≥15 years old diagnosed between 2001 and July 2012 only 15 (25%) were female. There was a highly statistically significant difference between the proportion who were female in the arthritis group versus the EM group (*X^2^* = 19.5, p<0.0001) and similarly versus the ACA group (*X^2^* = 22.1, p<0.0001).

As the case definition for Lyme arthritis used in the present study conceivably could have included seropositive patients with arthritis caused by a condition other than Lyme borreliosis, we also evaluated the gender distribution in the subgroup of these patients who had either a concomitant EM skin lesion or evidence of Lyme arthritis based on detection of *B. burgdorferi* sensu lato in joint fluid by culture or by polymerase chain reaction. In this subgroup of 22 patients (whose median age of 47 years was similar to the median age of 48.5 years for the entire group of Lyme arthritis cases), 5 (22.7%) were female, suggesting that misdiagnosis did not contribute to the male predominance among Lyme arthritis cases.

Among the 130 patients ≥15 years old with LNB diagnosed from October 2005 to July 2012, there was also a male predominance. In this group only 51 (39.2%) were females, which was significantly different compared with the EM group (*X^2^* = 19.1, p<0.0001) or the ACA group (*X^2^* = 17.6, p<0.0001). Although the proportion of females in the LNB group was greater than in the Lyme arthritis group, this difference did not reach statistical significance (*X^2^* = 2.7, p = 0.10). All of these comparisons were performed after correcting for differences in age.

## Discussion

The results of this study conducted at a medical center in Slovenia show that the number of females diagnosed with EM or ACA exceeded the number of males with these cutaneous manifestations of Lyme borreliosis. The opposite was found for non-cutaneous manifestations, where the number of males diagnosed with LNB or Lyme arthritis exceeded the number of females. A female predominance among patients with ACA is quite consistent among case series of ACA reported from other European countries [15]–[17]. There has also been a female predominance among EM cases in many case series of adult patients from other European countries [17]–[21], with an especially high proportion of females in patients with recurrent EM in Sweden [22], [23]. Similarly, a male predominance for LNB or Lyme arthritis in Europe is by no means unique to Slovenia [4], [24]–[30]. A particular strength of our study is that we had data on a large number of patients with different clinical manifestations of Lyme borreliosis who were diagnosed at a single medical center, thus avoiding confounding variables that may be present when comparisons are made between medical centers.

To our knowledge, this is the first report to document at the same medical center that the proportion of ACA patients who are female is substantially greater than those diagnosed with EM. The difference in the proportion who were females between patients with ACA and EM, however, can be explained almost entirely by differences in the ages of these patients. ACA is a late manifestation of Lyme borreliosis, and patients with ACA were about 15 years older than those with EM. The sex ratio in Slovenia is increasingly biased toward females in older age groups [12], and the difference between ACA and EM patients becomes marginal after controlling for this trend. The observed differences in the proportions of females with EM or ACA, compared with those with either LNB or Lyme arthritis, however, cannot be accounted for by the ages of the patient groups.

Although the patient groups in this study were not from identical time periods, this could only substantively influence the results if there were marked time-related shifts in the sex ratio of the general Slovenian population between 1990 and 2012. Census data, however, indicate that females accounted for 51.5%, 51.1% and 50.5% of the Slovenian population in 1990, 2000, and 2010 respectively, underscoring that there have been no major shifts in the sex ratio [12]. In addition, if we had limited our analysis of EM cases to the 5,049 cases (48%) occurring during the overlapping years of 2000–2009, the female preponderance was essentially unchanged at 58%. Thus, time-related changes in the sex ratio cannot account for the observed gender differences between cutaneous and non-cutaneous clinical manifestations of Lyme borreliosis.

What might account for the male predominance among patients with LNB and Lyme arthritis? One possibility is that strains of *B. garinii* and *B. burgdorferi* sensu stricto, the Lyme borrelial species thought to be most closely associated with LNB and Lyme arthritis respectively [1], are less likely to infect women than men. This would be plausible if the ticks infected with these species of Lyme borrelia were preferentially found in geographic areas frequented more by men than women, or alternatively, if men were more susceptible to developing infection with these species after being bitten by an infected tick. With either of these scenarios, one might expect, relative to *B. afzelii* infection, that there would be a lower proportion of women with EM who had a positive skin culture for *B. garinii* or for *B. burgdorferi* sensu stricto. Table 2 shows the available Slovenian data on gender for the patients with a positive culture of a skin biopsy sample of an EM skin lesion [31]–[35]. In support of these hypotheses, a higher percentage of patients with *B. afzelii* infection were female compared with those infected with *B. garinii* (62.2% vs. 53.3%, p = 0.06), and this difference was statistically significant when the rates were adjusted for the age of the patients (59.1% vs. 44.1%, p = 0.003). Indeed, after the age correction there was a male predominance for *B. garinii* infections. However, this pattern was not found for *B. burgdorferi* sensu stricto.

**Table 2:** Correlation between the Species of Lyme Borrelia Isolated from the Skin of Patients with Erythema Migrans and the Gender of Infected Slovenian Patients.

Species	Years When Cultured	References	Number of Female Patients with a Positive Culture (%)	Age of Females in Years Median (range)	Number of Male Patients with a Positive Culture (%)	Age of Males in Years Median (range)
*Borrelia afzelii*	1993, 1997, 2006, 2009	31323334	310 (62.2)	53 (15–85)	188 (37.8)	46 (16–79)
*Borrelia garinii*	1993–2007, 2009	3534	65 (53.3)	56 (22–79)	57 (46.7)	52 (20–83)
*Borrelia burgdorferi* sensu stricto	1993–2009	Unpublished data	13 (76.5)	55 (33–77)	4 (23.5)	39 (18–47)

Since LNB and Lyme arthritis presumably arise in most cases through hematogenous dissemination [36], it may be that the likelihood of spirochetemia with highly neurotropic strains of *B. garinii* [37]–[39], or with strains of *B. burgdorferi* sensu stricto with the strongest propensity to infect joints [37], is greater in males than females. Possible differences between females and males in the immunologic response to borrelia has been discussed by others in trying to explain the much greater likelihood of recurrent episodes of EM in females from Sweden compared with men [21], [23]. In the experience at the University Medical Center Ljubljana with patients found to have a positive blood culture for *B. garinii*, there were 12 females compared with 10 males; among patients with a positive blood culture for *B. burgdorferi* sensu stricto, there were 3 females and no males to date. Clearly the available data are too limited to exclude the possibility that gender might influence the risk of hematogenous dissemination with certain strains of *B. garinii* or *B. burgdorferi* sensu stricto.

Perhaps the explanation is purely behavioral. It could be argued that Slovenian men are simply less likely than Slovenian women to seek health care for EM skin lesions, and thus they become at higher risk for dissemination of Lyme borrelia to extracutaneous sites. Available data in Slovenia on utilization of health care indicate among individuals aged ≥20 years that women had more outpatient primary care visits in 2010 than men with a ratio of 1.3 visits for women to 1 for men [40]. A counter-argument against this hypothesis, however, is the clear female predominance among patients with ACA, which is also a late manifestation of Lyme borreliosis [1]. However, it may be that men are simply less likely than women in Slovenia to seek medical care for any kind of skin lesion including both EM and ACA.

The potential for biologic significance of gender in infectious diseases is not unique to Lyme borreliosis. For example, it has been previously demonstrated in a murine model of *Borrelia hermsii* infection that males have a significantly higher initial peak level of spirochetemia than females [41]. In another example, it has been observed in patients with *Coxiella burnetii* infection (Q fever) that men are 2.5 times more likely to be symptomatic than women [42], and experimental studies in mice have confirmed that sex hormones play a role in the pathophysiology of this infection [43].

In conclusion, in this study performed at a single medical center in Slovenia, patients with the cutaneous manifestations of Lyme borreliosis, EM and ACA, were predominantly female, while those with LNB or Lyme arthritis were predominantly men. This pattern of gender distribution has been noted in other [4], [15]–[30], but not all [21], [44]–[47], case series from Europe of these particular clinical manifestations of Lyme borreliosis. Elucidation of the mechanism(s) involved may provide useful insights into the pathogenesis of Lyme borreliosis in Europe.

## References

[1] Stanek G, Wormser GP, Gray J, Strle F (2012) Lyme borreliosis. Lancet 379: 461–473. 21903253 10.1016/S0140-6736(11)60103-7

[2] Bacon RM, Kugeler KJ, Mead PS (2008) Surveillance for Lyme disease – United States, 1992–2006. Morbidity Mortality Weekly Report (MMWR) 57 (SS10) 1–9. 18830214

[3] Wormser GP, Shapiro ED (2009) Implications of gender in chronic Lyme disease. J Womens Health 18: 831–834. 10.1089/jwh.2008.1193 PMC2913779 19514824

[4] Fulop B, Poggensee G (2008) Epidemiological situation of Lyme borreliosis in Germany: surveillance data from six eastern German states, 2002 to 2006. Parasitol Res 103: S117–S120. 19030893 10.1007/s00436-008-1060-y

[5] Mehnert WH, Krause G (2005) Surveillance of Lyme borreliosis in Germany, 2002 and 2003. Eurosurveillance 10: 83–85. 15879644

[6] Institute of Public Health of the Republic of Slovenia. Epidemiologic surveillance of communicable diseases in Slovenia in 2010. Ljubljana, 2011. Available: http://www.ivz.si. Accessed 2013 Mar 12.

[7] Centers for Disease Control and Prevention (CDC) website. Available: http://wwwn.cdc.gov/nndss/document/2012_Case%20Definitions.pdf. Accessed 2013 Mar 12.

[8] Wilske B, Schierz G, Preac-Mursic V, Weber K, Pfister HW, et al (1984) Serological diagnosis of erythema migrans disease and related disorders. Infection 5: 331–337. 10.1007/BF01651147 6392104

[9] Strle F, Stanek G (2009) Clinical manifestations and diagnosis of Lyme borreliosis. Curr Probl Dermatol 37: 51–110. 19367097 10.1159/000213070

[10] Marangoni A, Moroni A, Accardo S, Cevenini R (2008) *Borrelia burgdorferi* VlsE antigen for the serological diagnosis of Lyme borreliosis. Eur J Clin Microbiol Infect Dis 27: 349–354. 18197445 10.1007/s10096-007-0445-7

[11] Reiber H, Peter JB (2001) Cerebrospinal fluid analysis: disease-related data patterns and evaluation programs. J Neurol Sci 184: 101–122. 11239944 10.1016/s0022-510x(00)00501-3

[12] Statistical Office of the Republic of Slovenia website. Available: http://www.stat.si/eng/index.asp. Accessed 2013 Feb 28.

[13] Selvin S (1991) Statistical Analysis of Epidemiological Data. New York: Oxford University Press. pp. 375.

[14] Woolson RF, Bean JA (1982) Mantel-Haenszel statistics and direct standardization. Statistics in Medicine 1: 37–39. 7187081 10.1002/sim.4780010106

[15] Asbrink E (1993) Acrodermatitis chronica atrophicans. Clin Dermatol 11: 369–375. 8221518 10.1016/0738-081x(93)90092-q

[16] Brehmer-Andersson E, Hovmark A, Asbrink E (1998) Acrodermatitis chronica atrophicans: histopathologic findings and clinical correlations in 111 cases. Acta DermVenereol 78: 207–213. 10.1080/000155598441558 9602229

[17] Hulshof MM, Vandenbroucke JP, Nohlmans LMKE, Spanjaard L, Bavinck JNB, et al (1997) Long-term prognosis in patients treated for erythema chronicum migrans and acrodermatitis chronica atrophicans. Arch Dermatol 133: 33–37. 9006370

[18] Tibbles CD, Edlow JA (2007) Does this patient have erythema migrans? JAMA 297: 2617–2627. 17579230 10.1001/jama.297.23.2617

[19] Bennet L, Halling A, Berglund J (2006) Increased incidence of Lyme borreliosis in Southern Sweden following mild winters and during warm, humid summers. Eur J Clin Microbiol Infect Dis 25: 426–432. 16810531 10.1007/s10096-006-0167-2

[20] Marangoni A, Sambri V, Accardo S, Carvini F, Mondardini V, et al (2006) A decrease in the immunoglobulin G antibody response against VlsE protein of *Borrelia burgdorferi* sensu lato correlates with the resolution of clinical signs in antibiotic-treated patients with early Lyme disease. Clin Vaccine Immunol 13: 525–529. 16603623 10.1128/CVI.13.4.525-529.2006 PMC1459630

[21] Bennet L, Stjernberg L, Berglund J (2007) Effect of gender on clinical and epidemiologic features of Lyme borreliosis. Vector-Borne Zoonotic Dis 7: 34–41. 17417955 10.1089/vbz.2006.0533

[22] Bennet L, Berglund J (2002) Re-infection with Lyme borreliosis: A retrospective follow-up study in Southern Sweden. Scand J Infect Dis 34: 183–186. 12030390 10.1080/00365540110080070

[23] Jarefors S, Bennet L, You E, Forsberg P, Ekerfelt C, et al (2006) Lyme borreliosis reinfection: might it be explained by a gender difference in immune response. Immunology 118: 224–232. 16771857 10.1111/j.1365-2567.2006.02360.x PMC1782288

[24] Renaud I, Cachin C, Gerster JC (2004) Good outcomes of Lyme arthritis in 24 patients in an endemic area of Switzerland. Joint Bone Spine 71: 39–43. 14769519 10.1016/S1297-319X(03)00160-X

[25] Berglund J, Hansen BU, Eitrem R (1995) Lyme arthritis – a common manifestation in a highly endemic area in Sweden. J Rheumatol 22: 695–701. 7791166

[26] Pancewicz S, Popku J, Rutkowski R, Knaś M, Grygorczuk S, et al (2009) Activity of liposomal exoglycosidoses in serum and synovial fluid in patients with chronic Lyme and rheumatoid arthritis. Scand J Infect Dis 41: 584–589. 19513935 10.1080/00365540903036220

[27] Luczaj W, Moniuszko A, Rusak M, Pancewicz S, Zajkowska J, et al (2011) Lipid peroxidation products as potential bio-indicators of Lyme arthritis. Eur J Clin Microbiol Infect Dis 30: 415–422. 21057969 10.1007/s10096-010-1102-0

[28] Kirchner A, Koedel W, Fingerle V, Paul R, Wilske B, et al (2000) Up-regulation of matrix metalloproteinase-9 in the cerebrospinal fluid of patients with acute Lyme neuroborreliosis. J Neurol Neurosurg Psych 68: 368–371. 10.1136/jnnp.68.3.368 PMC1736835 10675223

[29] Van Burgel ND, Brandenburg A, Gerritsen HJ, Kroes ACM, Van Dam AP (2011) High sensitivity and specificity of the C6-peptide ELISA on cerebrospinal fluid in Lyme neuroborreliosis patients. Clin Microbiol Infect 17: 1495–1500. 21375653 10.1111/j.1469-0691.2011.03459.x

[30] Ljostad U, Skogvall E, Eikeland R, Midgard R, Skarpaas T, et al (2008) Oral doxycycline versus intravenous ceftriaxone for European Lyme neuroborreliosis: a multicentre, non-inferiority, double-blind, randomized trial. Lancet Neurol 7: 690–695. 18567539 10.1016/S1474-4422(08)70119-4

[31] Strle F, Nadelman RB, Cimperman J, Nowakowski J, Picken RN, et al (1999) Comparison of culture-confirmed erythema mirgans caused by *Borrelia burgdorferi* sensu stricto in New York state and by *Borrelia afzelii* in Slovenia. Ann Intern Med 130: 32–36. 9890847 10.7326/0003-4819-130-1-199901050-00006

[32] Logar M, Ružić-Sabljić E, Lotrič-Furlan S, Cimperman J, Jurca T, et al (2004) Comparison of erythema migrans caused by *Borrelia afzelii* and *Borrelia garinii* . Infection 32: 15–19. 15007737 10.1007/s15010-004-3042-z

[33] Cerar D, Cerar T, Ružić-Sabljić E, Wormser GP, Strle F (2010) Subjective symptoms after treatment of early Lyme disease. Am J Med 123: 79–86. 20102996 10.1016/j.amjmed.2009.05.011

[34] Stupica D, Lusa L, Ružić-Sabljić E, Cerar T, Strle F (2012) Treatment of erythema migrans with doxycycline for 10 days versus 15 days. Clin Infect Dis 55: 343–350. 22523260 10.1093/cid/cis402

[35] Strle F, Ružić-Sabljić E, Logar M, Maraspin V, Lotrič-Furlan S, et al (2011) Comparison of erythema migrans caused by *Borrelia burgdorferi* and *Borrelia garinii* . Vector Borne Zoonotic Dis 11: 1253–1258. 21612533 10.1089/vbz.2010.0230

[36] Wormser GP, Brisson D, Liveris D, Hanincová K, Sandigursky S, et al (2008) *Borrelia burgdorferi* genotype predicts the capacity for hematogenous dissemination during early Lyme disease. J Infect Dis 198: 1358–1364. 18781866 10.1086/592279 PMC2776734

[37] Brisson D, Baxamusa N, Schwartz I, Wormser GP (2011) Biodiversity of *Borrelia burgdorferi* strains in tissues of Lyme disease patients. PLoS One 6 (8) e22926. 21829670 10.1371/journal.pone.0022926 PMC3150399

[38] Panelius J, Rank A, Meri T, Seppala I, Meri S (2010) Expression and sequence diversity of the complement regulating outer surface protein E in *Borrelia afzelii* vs. *Borrelia garinii* in patients with erythema migrans or neuroborreliosis. Microbiol Pathogenesis 49: 363–368. 10.1016/j.micpath.2010.06.006 20603210

[39] Alitalo A, Meri T, Comstedt P, Jeffery L, Tornberg J, et al (2005) Expression of complement factor H binding immunoevasion proteins in *Borrelia garinii* isolated from patients with neuroborreliosis. Eur J Immunol 35: 3043–3053. 16208765 10.1002/eji.200526354

[40] Institute of Public Health of the Republic of Slovenia website. Available: ZUBSTAT http://www.ivz.si/. Accessed 2012 Nov 10.

[41] Benoit VM, Petrich A, Alugupalli KR, Marty-Roix R, Moter A, et al (2010) Genetic control of the innate immune response to *Borrelia hermsii* influences the course of relapsing fever in inbred strains of mice. Infect Immun 78: 586–594. 19995898 10.1128/IAI.01216-09 PMC2812184

[42] Raoult D, Tissot-Dupont H, Foucault C, Gouvernet J, Fournier PE, et al (2000) Q fever 1985–1998. Clinical and epidemiologic features of 1,383 infections. Medicine 79: 109–123. 10771709 10.1097/00005792-200003000-00005

[43] Leone M, Honstettre A, Lepidi H, Capo C, Bayard F, et al (2004) Effect of sex on *Coxiella burnetii* infection: protective role of 17 beta-estradiol. J Infect Dis 189: 339–345. 14722900 10.1086/380798

[44] Huaux JP, Bigaignon G, Stadtsbaeder S, Zangerle PF, De Deuxchaisnes CN (1988) Pattern of Lyme arthritis in Europe: report of 14 cases. Ann Rheum Dis 47: 164–165. 3355251 10.1136/ard.47.2.164 PMC1003470

[45] Pietruczuk A, Swierzbinska R, Pancewicz S, Pietruczuk M, Hermanowska-Szpakowicz T (2006) Serum levels of interleukin-18 (IL-18), interleukin 1-beta (IL-1beta), its soluble receptor sIL-1RII and C-reactive protein (CRP) in patients with Lyme arthritis. Infection 34: 158–162. 16804660 10.1007/s15010-006-5013-z

[46] Karkkonen K, Stiernstedt SH, Karlsson M (2001) Follow-up of patients treated with oral doxycycline for Lyme neuroborreliosis. Scand J Infect Dis 33: 259–262. 11345216 10.1080/003655401300077225

[47] Ciceroni L, Ciarrochi S, Ciervo A, Mondarini V, Guzzo F, et al (2001) Isolation and characterization of *Borrelia burgdorferi* sensu lato strains in an area of Italy where Lyme borreliosis is endemic. J Clin Microbiol 39: 2254–2260. 11376066 10.1128/JCM.39.6.2254-2260.2001 PMC88120

